# Sub-Chronic Stress Exacerbates the Pro-Thrombotic Phenotype in BDNF^Val/Met^ Mice: Gene-Environment Interaction in the Modulation of Arterial Thrombosis

**DOI:** 10.3390/ijms19103235

**Published:** 2018-10-19

**Authors:** Leonardo Sandrini, Alessandro Ieraci, Patrizia Amadio, Fabrizio Veglia, Maurizio Popoli, Francis S. Lee, Elena Tremoli, Silvia Stella Barbieri

**Affiliations:** 1 Dipartimento di Scienze Farmacologiche e Biomolecolari, Università degli Studi di Milano, 20133 Milan, Italy; leonardo.sandrini@unimi.it (L.S.); alessandro.ieraci@unimi.it (A.I.); maurizio.popoli@unimi.it (M.P.); 2 Centro Cardiologico Monzino IRCCS, 20138 Milan, Italy; patrizia.amadio@ccfm.it (P.A.); fabrizio.veglia@ccfm.it (F.V.); elena.tremoli@ccfm.it (E.T.); 3 Department of Psychiatry, Weill Cornell Medical College of Cornell University, New York, NY 10065, USA; fslee@med.cornell.edu

**Keywords:** BDNF Val66Met, thrombosis, sub-chronic stress

## Abstract

Brain-Derived Neurotrophic Factor (BDNF) Val66Met polymorphism has been associated with increased susceptibility to develop mood disorders and recently it has been also linked with cardiovascular disease (CVD). Interestingly, stressful conditions unveil the anxious/depressive-like behavioral phenotype in heterozygous BDNFVal66Met (BDNF^Val/Met^) mice, suggesting an important relationship in terms of gene-environment interaction (GxE). However, the interplay between stress and BDNF^Val/Met^ in relation to CVD is completely unknown. Here, we showed that BDNF^Val/Met^ mice display a greater propensity to arterial thrombosis than wild type BDNF^Val/Val^ mice after 7 days of restraint stress (RS). RS markedly increased the number of leukocytes and platelets, and induced hyper-responsive platelets as showed by increased circulating platelet/leukocyte aggregates and enhanced expression of P-selectin and GPIIbIIIa in heterozygous mutant mice. In addition, stressed BDNF^Val/Met^ mice had a greater number of large and reticulated platelets but comparable number and maturation profile of bone marrow megakaryocytes compared to BDNF^Val/Val^ mice. Interestingly, RS led to a significant reduction of BDNF expression accompanied by an increased activity of tissue factor in the aorta of both BDNF^Val/Val^ and BDNF^Val/Met^ mice. In conclusion, we provide evidence that sub-chronic stress unveils prothrombotic phenotype in heterozygous BDNF Val66Met mice affecting both the number and functionality of blood circulating cells, and the expression of key thrombotic molecules in aorta. Human studies will be crucial to understand whether this GxE interaction need to be taken into account in risk stratification of coronary artery disease (CAD) patients.

## 1. Introduction

Increasing evidence suggests that environmental stressors, both physical and psychosocial, affect not only the onset and progression of central nervous system (CNS) disorders but also contribute to increase risk, incidence, and severity of cardiovascular disease (CVD) and the response to therapy [1].

The hyperactivation of the hypothalamic–pituitary–adrenal (HPA) axis and stimulation of the sympathetic adrenomedullary system under stressful conditions lead to release of cortisol and catecholamines in the circulatory system [2]. These stress hormones play a key function in the modulation of acute stress adaptation but are responsible of pathological dysregulation when stress exposure persists. In the CNS, they affect the expression of different genes and molecules involved in brain plasticity such as neurotrophins [3]. Moreover, in the periphery they stimulate megakaryo- and thrombo-poiesis, induce platelet activation [4,5] and endothelial dysfunction, enhance coagulation pathway activation and reduce fibrinolysis [6] predisposing to onset and progression of CVD [7] and arterial thrombosis [8]. However, stressors do not affect everyone in the same way. Some individuals are more susceptible and express inappropriate pathological responses to stressors; in contrast, others, often called resilient individuals, perceive adversity minimally and develop adaptive physiological and psychological responses [9,10]. In this context, genetic and epigenetic alterations may influence vulnerability or resiliency to stressor(s), predisposing to or protecting individuals with different diseases, including CNS disorders and CVD [11,12].

Brain-derived neurotrophic factor (BDNF), a neurotrophin involved in neural development [13] and adult brain plasticity [14], has been associated with stress response, depression and anxiety [3,15]. Indeed, BDNF regulates many functions carried out by the HPA axis [16], and the expression of BDNF is dramatically affected under stressful conditions [17,18]. 

Interestingly, the BDNF Val66Met single nucleotide polymorphism (SNP), which reduces the activity-dependent release of BDNF and affects protein and mRNA levels [19,20,21,22], has been associated with an increased susceptibility to develop cognitive and psychiatric disorders [23,24].

As other gene polymorphisms related to mood disorders [12], this SNP has been recently associated with CVD [25,26]. In particular, BDNF Val66Met polymorphism is associated to propensity for arterial thrombosis in a cohort of patients with acute myocardial infarction [27]. Consistent with these human data, homozygous BDNF^Met/Met^ mice display a prothrombotic phenotype with alterations in coagulation pathways and platelet activation [27]. 

The emerging hypothesis that genetic alterations predisposes individuals to vulnerability under stressful conditions is well supported by the results that heterozygous BDNF Val66Met mice display anxiety- and depression-like behavior phenotypes only after exposure to a paradigm of sub-chronic stress [28]. 

However, few studies have examined the interaction of gene-environment (GxE) in CVD [12]. In particular, evidence related to the interaction between BDNF^Val/Met^ and stress in the onset of CVD is not available. In this study, taking advantage of the BDNF Val66Met knock-in mouse model, we have investigated the response of BDNF^Val/Met^ mice to stress in relation to arterial thrombosis. 

## 2. Results

### 2.1. Sub-Chronic Restraint Stress (RS) Induced Activation of the Hypothalamic-Pituitary-Adrenal Axis and Altered Body Weight

To evaluate whether 7 days of sub-chronic restraint stress (RS) induced a different HPA axis response in BDNF^Val/Val^ and BDNF^Val/Met^ mice, we measured the weight of adrenal glands, as a key step of HPA axis activation.

**Figure 1:**
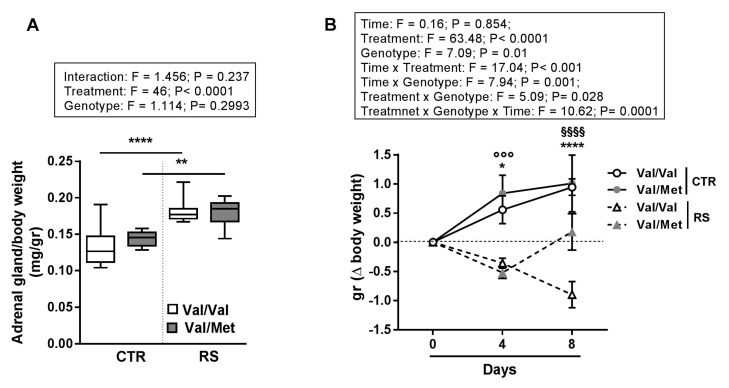
Effect of sub-chronic stress on BDNF^Val/Val^ and BDNF^Val/Met^ mice. Sub-chronic stress was induced by restraint stress (RS) treatment for 7 days, 2 h/day. (**A**) Adrenal gland weight to body weight ratio (**B**) Delta (Δ) body weight of mice at day 4 and day 8 of stressed (RS) and not stressed (CTR) BDNF^Val/Val^ and BDNF^Val/Met^ mice. Data are expressed as mean ± SEM. *n* = 8 mice/group. Two-way ANOVA and three-way ANOVA with repeated measures followed by Bonferroni post hoc analysis, *p* values were obtained by using log-transformed variables. * *p* < 0.05, ** *p* < 0.01, **** *p* < 0.005 BDNF^Val/Val^ (CTR) vs. BDNF^Val/Val^ stressed (RS) mice, °°° *p* < 0.005 BDNF^Val/Met^ (CTR) vs. BDNF^Val/Met^ stressed (RS) mice and ^§§§§^ *p* < 0.00015 BDNF^Val/Val^ stressed (RS) vs. BDNF^Val/Met^ stressed (RS) mice. For each box-plot, the center line illustrates the median and box limits indicate the 25th and 75th percentiles.

Adrenal gland weight was greater in both BDNF^Val/Val^ and BDNF^Val/Met^ stressed mice compared to controls and no differences were found between the genotypes, suggesting a comparable level of stress-response (Figure 1A).

Interestingly, while the weight of BDNF^Val/Val^ stressed mice decreased progressively along 7 days of RS, the weight of BDNF^Val/Met^ mice decreased during the first 4 days of RS and returned to basal levels during the following days (Figure 1B).

### 2.2. RS Predisposed BDNF^Val/Met^ Mice to Thrombosis

To understand whether the interaction between stress and BDNF polymorphism resulted in a prothrombotic phenotype, we investigated the effect of sub-chronic RS in BDNF^Val/Val^ and BDNF^Val/Met^ mice focusing on arterial thrombus formation in vivo after topical application of FeCl_3_ to exposed carotid.

**Figure 2:**
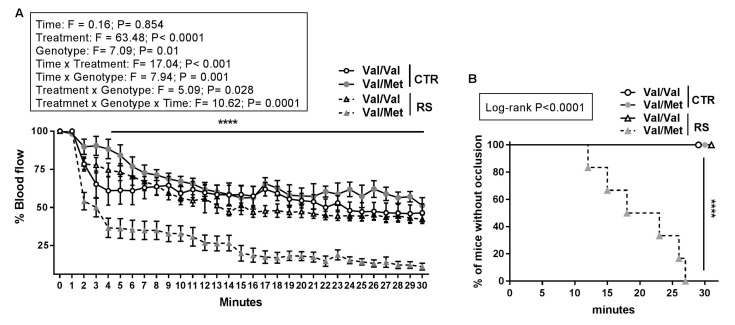
Effect of restraint stress on arterial thrombosis in BDNF^Val/Val^ and BDNF^Val/Met^ mice. Arterial thrombosis was induced by topical application of FeCl_3_ to carotid artery in mice. (**A**) Blood flow in the carotid arteries of stressed (RS) and not stressed (CTR) BDNF^Val/Val^ and BDNF^Val/Met^ mice groups. *n* = 6 mice/group. Data shown are mean ± SEM. Three-way ANOVA with repeated measures followed by Bonferroni post hoc analysis, *p* values were obtained by using log-transformed variables. (**B**) Kaplan-Meier curve representing percentage of mice without occlusion through time. *n* = 6 mice/group. *p* values were obtained by analysing Kaplan-Meier curves by Log-Rank tests. **** *p* < 0.001.

RS reduced about 70% of the blood flow in BDNF^Val/Met^ mice after 4 min from FeCl_3_ application, whether the blood flow of BDNF^Val/Val^ stressed mice was only slightly affected (Figure 2A). Similarly, a partial occlusion of carotid artery was observed in the BDNF^Val/Val^ and BDNF^Val/Met^ not-stressed (CTR) mice without reaching a stable occlusion (Figure 2A). In line with these data, total occlusion (flow reduction >90%) was reached only in stressed BDNF^Val/Met^ mice after an average time of 20 min (Figure 2B). 

Overall, these data show that heterozygous Met allele *per se* is not sufficient to predispose to thrombosis and that the sub-chronic stressful condition triggers the pro-thrombotic phenotype of BDNF^Val/Met^ mice.

### 2.3. RS Increased Circulating Blood Cell Number and Platelet Activation State 

In our experimental setting, there was a main genotype effect between BDNF^Val/Val^ and BDNF^Val/Met^ mice in terms of circulating blood cells and platelet function.

**Figure 3:**
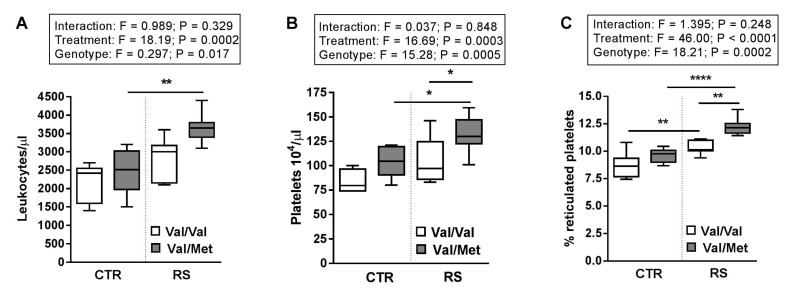
Circulating blood cell numbers are increased by restraint stress. (**A**) Numbers of circulating leukocytes, (**B**) platelets were counted, and (**C**) the percentage of reticulated platelets were analysed by flow cytometry in stressed (RS) and not stressed (CTR) BDNF^Val/Val^ and BDNF^Val/Met^ mice. *n* = 6 mice/group. Two-way ANOVA followed by Bonferroni post hoc analysis, *p* values were obtained by using log-transformed variables. * *p* < 0.05, ** *p* < 0.01 and **** *p* < 0.001. For each box-plot, the center line illustrates the median and box limits indicate the 25th and 75th percentiles.

RS increased the number of leukocytes (Figure 3A) and platelets (Figure 3B), as well as the percentage of reticulated platelets (Figure 3C) in both BDNF^Val/Val^ and BDNF^Val/Met^ mice compared to not stressed mice (CTR). Remarkably, the effect of RS was greater in BDNF^Val/Met^ platelets than in BDNF^Val/Val^ (Figure 3B,C).

**Figure 4:**
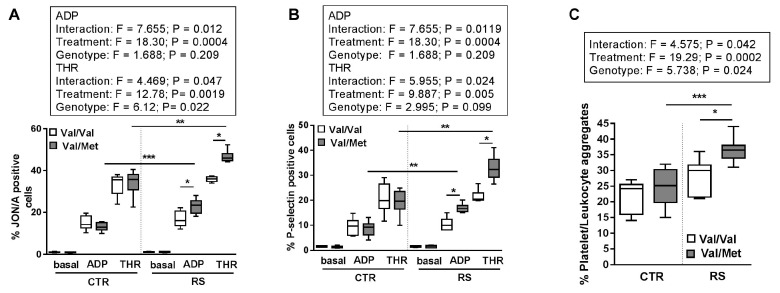
Effect of restraint stress on platelet activation in BDNF^Val/Val^ and BDNF^Val/Met^ mice. Flow cytometry analyses of: (**A**) GPIIbIIIa activation (JON/A-PE antibody); (**B**) P-selectin expression in washed platelets at basal condition or after exposure to ADP (5 µM) or thrombin (THR 0.05 U/mL); and percentage of (**C**) platelet/leukocytes in whole blood isolated from stressed (RS) and not stressed (CTR) BDNF^Val/Val^ and BDNF^Val/Met^ mice. *n* = 6 mice/group. Two-way ANOVA followed by Bonferroni post hoc analysis, *p* values were obtained by using log-transformed variables. * *p* < 0.05, ** *p* < 0.01 and *** *p* < 0.005. For each box-plot, the center line illustrates the median and box limits indicate the 25th and 75th percentiles.

In addition, while platelets from BDNF^Val/Val^ stressed mice displayed similar activation of integrin αIIbβ3 (GPIIbIIIa) (Figure 4A) and expression of P-selectin (Figure 4B) in response to thrombin (THR) and ADP compared to mice not exposed to RS, platelets from BDNF^Val/Met^ stressed mice showed a marked hyper-reactivity (Figure 4A,B).

Finally, RS enhanced the formation of the platelet/leukocyte aggregates in response to ADP in both BDNF^Val/Val^ and BDNF^Val/Met^ mice. However, the percentage of platelet/leukocyte aggregates increased more markedly in stressed BDNF^Val/Met^ mice compared to both BDNF^Val/Met^ mice not exposed to RS and BDNF^Val/Val^ mice exposed to RS (Figure 4C).

### 2.4. RS Increased Bone-Marrow Megakaryocytes Number without Affecting Their Maturation State

To understand if the change in platelet number detected under stressful conditions was the consequence of an alteration of megakaryopoiesis process, immunocytochemistry analysis of the femur bone marrow was carried out. 

**Figure 5:**
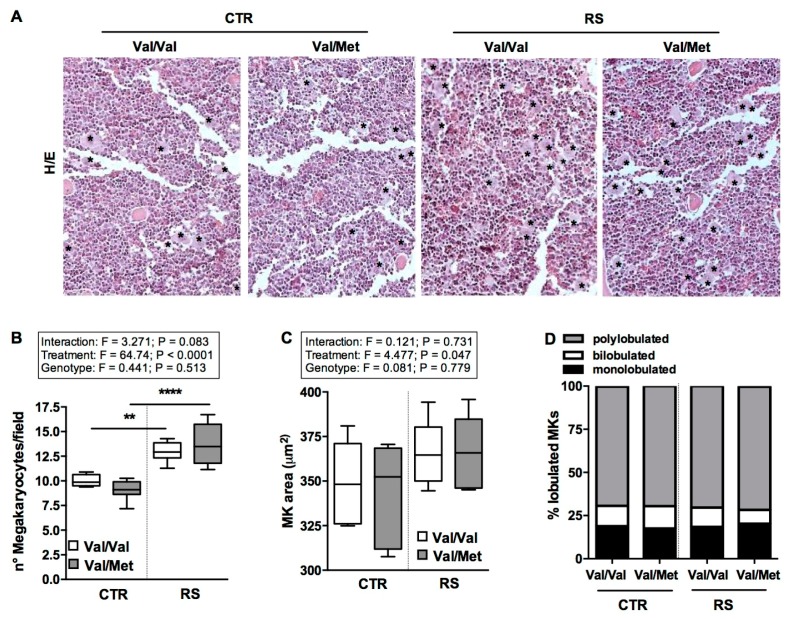
Effect of restraint stress on bone-marrow megakaryocytes. (**A**) Hematoxylin and Eosin (H&E) staining of bone marrow from stressed (RS) and not stressed (CTR) BDNF^Val/Val^ and BDNF^Val/Met^ mice. Asterisks indicate MKs. (**B**) Quantification of panel (**A**) expressed as megakaryocytes per field (40× magnification). Analysis of (**C**) area and of (**D**) nuclear complexity in megakaryocytes. *n* = 8 mice/group. Two-way ANOVA followed by Bonferroni post hoc analysis, *p* values were obtained by using log-transformed variables. ** *p* < 0.01 and **** *p* < 0.001. For each box-plot, the center line illustrates the median and box limits indicate the 25th and 75th percentiles.

Analysis of femur bone marrow sections evidenced that RS enhanced the number of megakaryocytes (MKs) in both BDNF^Val/Val^ and BDNF^Val/Met^ mice (Figure 5A,B). Nevertheless, MKs displayed similar dimensions (Figure 5C) and nuclear complexity (Figure 5D) with comparable percentage of mononucleated, binucleated, or polynucleated cells among all the analyzed groups.

### 2.5. RS Altered the Expression of Tissue Factor and BDNF but Not the Expression of Sirt1

It is well known that stress modulates tissue-factor (TF) activity [8], a key regulator of thrombus formation. Therefore, we examined expression and activity of TF in carotid artery tissues.

**Figure 6:**
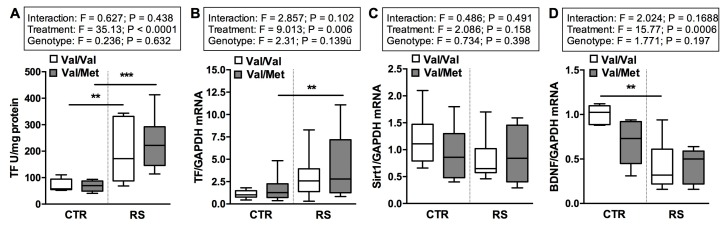
Impact of restraint stress on Tissue Factor, BDNF and Sirt1 expression in arterial tissue. (**A**) Tissue Factor (TF) activity and (**B**) TF, (**C**) Sirt1, (**D**) BDNF mRNA levels in arterial tissue of stressed (RS) and not stressed (CTR) BDNF^Val/Val^ and BDNF^Val/Met^ mice. *n* = 8 mice/group. Two-way ANOVA followed by Bonferroni *post hoc* analysis, *p* values were obtained by using log-transformed variables. ** *p* < 0.01, and *** *p* < 0.005. For each box-plot, the center line illustrates the median and box limits indicate the 25th and 75th percentiles.

Interestingly, while RS increased the activity of TF in carotid tissue (Figure 6A) of both BDNF^Val/Val^ and BDNF^Val/Met^ mice, the exposure to this stressful condition enhanced TF mRNA expression only in BDNF^Val/Met^ mice (Figure 6B). Comparable levels of TF expression and activity were detected in the control groups (Figure 6A,B).

Then, we measured the expression of Sirt1, an important modulator of TF and arterial thrombosis [29,30]. However, we did not found significant changes in terms of Sirt1 mRNA expression in arterial tissues among all the groups analyzed (Figure 6C). 

Of note, BDNF expression was comparable in control BDNF^Val/Met^ and BDNF^Val/Val^ mice, and although RS affected its expression in both genotypes this reduction was higher in BDNF^Val/Val^ mice (Figure 6D).

## 3. Discussion

Starting from the hypothesis that many multifactorial pathologies, including CVD, may result from the interaction between genes and environmental (GxE) factors and that some genetic variants, including BDNF Val66Met, are susceptible to stressors [12,15,31], we provide evidence that sub-chronic stress interacts with BDNF Val66Met polymorphism to unveil prothrombotic phenotype in a murine model. The major finding of this study is that seven days of RS, already reported to promote anxiety/depressive-like phenotype in heterozygous BDNF Val66Met (BDNF^Val/Met^) mice [28], are sufficient to exacerbate arterial thrombosis in BDNF^Val/Met^ mice and enhance platelet activation and coagulation pathway.

The dysfunctions in adrenergic and HPA axis observed in Met allele carriers [32] might explain their propensity to anxiety and depressive related disorder [28] and concomitantly to the development of a prothrombotic phenotype, observed here. Indeed, it is well known that the activation of HPA axis under stressful condition promotes dysregulation of both norepinephrine [2] and coagulation system (e.g., tissue factor and the plasminogen activation inhibitor-1) [33,34], and affects platelet number [4,5] and functionality [5,8,35,36,37,38], triggering the prothrombotic state. In addition, the significant volumetric reduction of the hippocampus [39] and the hyperactivation of amygdala [40] detected in humans carrying at least one Met allele and also observed in subjects under stressful conditions and neuropsychiatric disorders [41,42], support this hypothesis. Indeed, hippocampal neurons exert an inhibitory effect on the activation of the HPA axis, whereas the activity of the amygdala exerts a significant excitatory effect on the axis. Interestingly, the increased activity of amygdala has been recently associated with increased risk of CVD events and with enhanced bone marrow activity [43]. We show here that seven days of RS are sufficient to enhance the number of bone marrow MKs, circulating leukocytes and platelets and to induce platelet activation in BDNF^Val/Val^ mice, according to previously data obtained from both human and animal models [4,5,35]. These effects disproportionately increased in stressed BDNF^Val/Met^ mice predisposing them to thrombosis. Interestingly, all the alterations observed here in stressed heterozygotes BDNF^Val/Met^ mice recapitulate the phenotype observed in non-stressed BDNF^Met/Met^ homozygous mice [27].

Recently, Stämpfli et al. have shown that acute RS, although inducing arterial thrombosis in mice, did not affect activity and expression of TF, the principal activator of blood coagulation, in the vessel wall [8]. Here, we provide evidence that in wild type BDNF^Val/Val^ mice sub-chronic RS increases TF activity without modifying mRNA, suggesting that a protracted stress is able to modulate TF in a post-transcriptional manner. Remarkably both TF expression and activity were positively modulated in BDNF^Val/Met^ mice under sub-chronic stress, supporting the hypothesis of BDNF gene vulnerability. However, sub-chronic stress did not modify the expression of Sirt1, a modulator of TF expression and of arterial thrombosis, as previously shown in BDNF^Met/Met^ mice [27], in all experimental groups considered. Further experiments will be performed to understand which pathways are involved in the modulation of TF induced by protracted stress in order to unveil new possible targets that limit cardiovascular complications associated with environmental factors.

Interestingly, both BDNF Met allele variant and stressful conditions are associated with lower expression of BDNF in the central nervous system [28,44]. However, no data are available concerning the impact of this polymorphism and/or stress in other tissues. We show that BDNF mRNA levels are only slightly reduced in the arterial tissue of BDNF^Val/Met^ mice compared to BDNF^Val/Val^. In addition, although sub-chronic RS induced a decreasing trend in BDNF expression in both BDNF^Val/Met^ and BDNF^Val/Val^, the reduction was significant only in BDNF^Val/Val^ suggesting that the reduction of BDNF in arterial tissue is possible only to a certain threshold level, supporting its fundamental role in the vascular physiology [45,46,47].

Finally, our data, showing a weight reduction in stressed BDNF^Val/Val^ mice along the experimental time, are consistent with a previous study [48]. The weight loss observed during the stress exposure is potentially due initially to an early decrease in food intake and subsequently to an increased in energy expenditure and in body temperature [48]. In contrast, in BDNF^Val/Met^ mice we observed a weight loss only in the first few days of RS, followed by a surprising gain of weight, which returns to basal levels at the eighth day. This trend might be explained by the critical role of BDNF in the regulation of food intake and body weight control [49]. Indeed, BDNF is an anorexigenic factor [49], and low levels of BDNF in hippocampus and dorsal-vagal complex [50] as well as mutation in the BDNF gene [19,51,52,53] are associated with hyperphagia, weight gain and obesity. On these premises, we hypothesize that sub-chronic stress unmasks the hyperphagic phenotype in BDNF^Val/Met^ mice balancing the normal initial weight loss by enhancing food intake.

## 4. Materials and Methods

### 4.1. Mice

All experiments have been performed in adult male BDNF^Val/Met^ mice and littermate BDNF^Val/Val^ mice (3–4 months old) derived from BDNF^Val/Val^ × BDNF^Val/Met^ crossed parents [19]. All experiments were approved by the National Ministry of Health-University of Milan Committee and of DGSA (12/2012). Surgical procedures were performed in mice anesthetized with ketamine chlorhydrate (75 mg/kg; Intervet, Segrate, Milan, Italy) and medetomidine (1 mg/kg; Virbac, Milan, Italy). Mice were weighted and sacrificed 24 h after the last session of restraint stress RS. Tissues and blood were collected and adrenal glands were weighted.

### 4.2. Restraint Stress (RS) Procedure

Sub-chronic stress was induced by restraint stress (RS) test performed as previously described [28]. Briefly, BDNF^Val/Val^ and BDNF^Val/Met^ mice were divided randomly into stressed (RS) and control (CTR) groups. RS was performed daily for 2 h for 7 consecutive days, in well ventilated polypropylene restrainers without access to food and water. At the end of the stress session, mice were returned to their home cage. CTR mice were handled for 2 min and then returned to home cage.

### 4.3. Arterial Thrombosis Model

Experimental arterial thrombosis was induced as previously described [54]. Left carotid artery of anesthetized mice was dissected free and placed in the probe (model 0.7 V, Transonic System, Ithaca, NY, USA) connected to transonic flowmeter (TransonicT106). After blood flow stabilization (baseline flow constant for 7 min at least 0.8 mL/s), a 1 × 1 mm strip of filter paper (Whatman N°1) soaked with FeCl_3_ (10% solution; Sigma-Aldrich, Saint Louis, MO, USA) was applied over the carotid artery. After 3 min, the filter paper was removed, the carotid artery washed with PBS, and the flow recorded for 30 min. An occlusion was considered to be total and stable when the flow was reduced by >90% from baseline until the 30 min observation time, with the flow during this period not changing by more than 1% from baseline per second.

### 4.4. Whole Blood Counts

Blood was collected into 3.8% sodium citrate (1:10 *vol*:*vol*) from anesthetized mice by cardiac venipuncture, and white blood cells and platelet were counted optically [29].

### 4.5. Platelet–Leukocyte Aggregate Analysis

Platelet/monocyte and platelet/neutrophil aggregates were analysed as previously described [5]. Briefly, citrated blood was stimulated where indicated with 5 µM ADP for 5 min and red blood cells were lysed by FACS Lysing solution; samples were stained with the anti-CD45, anti-CD41, and anti-CD14 or anti-Lys6G and analysed by flow FACS “Novocyte 3000”. A minimum of 5000 events was collected in the CD14^+^ or Lys6G^+^ gate.

### 4.6. Platelet Studies

Washed platelets (WPs) were obtained from platelet-rich plasma (PRP), isolated following centrifugation at 100× *g* for 10 min of citrated blood as previously described [27], with serial centrifugation and addition of 0.2 mM PGI_2_ and 0.01 mg/L apyrase. Platelet pellets were resuspended in HEPES-Tyrode’s buffer (137 mM NaCl, 20 mM HEPES, 5.6 mM glucose, 0.35% bovine serum, 1 mM MgCl_2_, 2.7 mM KCl, and 3.3 mM NaH_2_PO_4_). 25 μL of WPs (5 × 10^4^/μL in HEPES-Tyrode’s buffer supplemented with 1 mM CaCl_2_) was mixed with a saturating concentration of PE-conjugated JON/A antibody, raised against the activated form of GPIIbIIIa (αIIβΙII integrin), or with anti-CD62P and FITC-conjugated antibody and the mixture reacted with different concentrations of ADP or thrombin for 15 min at room temperature. The reaction was stopped by 400 μL ice-cold PBS, and samples were analysed within 30 min. Platelets were identified by forward and side scatter distribution and by anti-CD41 positivity.

Reticulated platelets were identified by the thiazole orange method [55]: 10 μL of PRP was incubated with 390 μL of thiazole orange or PBS as control and anti-CD41 at room temperature for 10 min, in the dark. Immediately after incubation, samples were analysed by flow cytometry collecting 10,000 CD41-positive events; the percentage of RP was recorded, and the absolute number of RP was calculated by multiplying by the platelet count.

### 4.7. Quantitative Real-Time Polymerase Chain Reaction (RT-qPCR)

Total cellular RNA was isolated from arterial tissue with TRIzol Reagent according to the manufacturer’s instructions. One µg of RNA was reverse-transcribed using iScript™ cDNA Synthesis Kit. RT-qPCR was then carried out to detect Tissue Factor (TF), Sirt1, BDNF, and GAPDH used for normalized. The primers used for mice were: *TF*: forward 5′-CGGGTGCAGGCATTCCAGAG-3′, reverse 5′-CTCCGTGGGACAGAGAGGAC-3′; *SIRT1*: forward 5′-AGCAGGTTGCAGGAATCCAA-3′, reverse 5′-CACGAACAGCTTCACAATCAACTT-3′; BDNF: forward 5′-TCGTTCCTTTCGAGTTAGCC-3′, reverse 5′-TTGGTAAACGGCACAAAAC-3′ and *GAPDH:* forward 5′-CGTGCCGCCTGGAGAAACC-3′, reverse 5′-TGGAAGAGTGGGAGTTGCTGTTG-3′. Samples of cDNA were incubated in 15 µL IQ Supermix containing BDNF, TF, Sirt1 or GADPH primers and fluorescent dye SYBR Green (Bio-Rad Laboratories, Segrate, Milan, Italy). RT-qPCR was carried out in triplicate for each sample on the CFX Connect real-time System (Bio-Rad Laboratories, Segrate, Milan, Italy). Gene expression was analyzed using parameters available in CFX Manager Software 3.1 (Bio-Rad Laboratories, Segrate, Milan, Italy).

### 4.8. Measurement of TF Activity in Aortic Tissue

Before assay, samples were lysed with 15 mM *n*-Octyl-B-d-glucopyranoside lysis buffer at 37 °C for 10 min, sonicated at 20 kHz for 20 s and diluted with 25 mM HEPES saline. The total protein concentrations of carotid artery homogenates was determined using the Bradford method. 40 µL of carotid artery homogenate (1.25 μg/uL), or vehicle were mixed with 40 µL citrated pooled wild-type mouse plasma and 40 µL CaCl_2_ (final concentration 5 mM), and procoagulant activity was quantified by a one-stage plasma recalcification time assay [56]. Clotting times were expressed in relative units/µg protein based on a standard curve of serially diluted human thromboplastin preparation. Plasma deficient of Factor VIIa as well as preincubation with specific TF-neutralizing antibody were used to demonstrate TF dependence of procoagulant activity.

### 4.9. Bone Marrow Histology and MK Analyses

Immunocytochemistry and analysis of megakaryocytes (MKs) was performed on bone marrow (BM) as previously described [5]. Tissues were fixed overnight in 4% formalin, embedded in paraffin, cut at 3 μm, and mounted on polarized slides. BM samples were decalcified in 10% EDTA, pH 8 for 10 days before paraffin embedding. The number and area of MKs were evaluated in hematoxylin and eosin stained sections by counting 5–7 40× microscopic fields for each tissue sample digitalized by a Zeiss Axioskop (Carl Zeiss, Milan, Italy) equipped with an intensified charge-coupled device (CCD) camera system (Photometrics, Tucson, AZ, USA).

### 4.10. Statistical Analysis

Statistical analyses were performed with GraphPad Prism 7.0 and SAS versus 9.4 software (SASA Institute, Cary, NC, USA). Data were analyzed by 2-way or 3-way ANOVA with or without repeated measures for main effects of treatment and time or stimuli as reported in every graph, followed by a Bonferroni post hoc analysis as appropriate. Kaplan-Meier curves were analyzed by Log-Rank tests. Having a skewed distribution, the variables were included into the analyses after logarithmic transformation. Values of *p* < 0.05 were considered statistically significant. Data are expressed as mean ± SEM. For each box-plot, the center line illustrates the median, and box limits indicate the 25th and 75th percentiles.

## 5. Conclusions

In conclusion, we provide evidence that sub-chronic stress unveils a prothrombotic phenotype in heterozygous BDNF^Val/Met^ mice affecting the number and functionality of blood circulating cells, and the expression of key thrombotic molecules in arterial tissue. Human studies will be crucial to understand whether this gene-environment interaction needs to be taken into account in the risk stratification of coronary artery disease (CAD) patients. The identification of patients carrying “vulnerable genes” will be useful to identify new strategies to prevent and treat CAD.

## Abbreviations

BDNF	Brain-Derived Neurotrophic Factor
CNS	Central Nervous System
CVD	Cardiovascular Disease
GxE	Gene-environment Interaction
HPA	Hypothalamic–Pituitary–Adrenal Axis
RS	Restraint Stress
